# Impact of E-liquid Packaging on Vaping Product Perceptions Among Youth in England, Canada, and the United States: A Randomized Online Experiment

**DOI:** 10.1093/ntr/ntad144

**Published:** 2023-08-05

**Authors:** Erikas Simonavičius, Katherine East, Eve Taylor, Matilda Nottage, Jessica L Reid, Deborah Arnott, Laura Bunce, Ann McNeill, David Hammond

**Affiliations:** Department of Addictions, Institute of Psychiatry, Psychology and Neuroscience, King’s College London, London, UK; Department of Addictions, Institute of Psychiatry, Psychology and Neuroscience, King’s College London, London, UK; Department of Addictions, Institute of Psychiatry, Psychology and Neuroscience, King’s College London, London, UK; Department of Addictions, Institute of Psychiatry, Psychology and Neuroscience, King’s College London, London, UK; School of Public Health Sciences, University of Waterloo, Waterloo, Ontario, Canada; Action on Smoking and Health, London, UK; Action on Smoking and Health, London, UK; Department of Addictions, Institute of Psychiatry, Psychology and Neuroscience, King’s College London, London, UK; School of Public Health Sciences, University of Waterloo, Waterloo, Ontario, Canada

## Abstract

**Introduction:**

Vaping is not risk-free but can help those who smoke to reduce harm to health and stop smoking. However, packaging of vaping products, including e-liquids, appeals to youth and might facilitate vaping among nicotine-naïve people. Standardized packaging of vaping products could moderate the appeal of vaping among youth. This study assessed how youth interest in trying and perceived health harms of using e-liquids are associated with branded or standardized (white or olive) e-liquid packaging with different nicotine levels displayed.

**Aims and Methods:**

A between-subject experiment with three packaging and two nicotine level conditions included youth (*n* = 13801) aged 16 to 19 from England, Canada, and the United States as a part of a cross-sectional online survey in August–September 2021. Participants’ interest in trying and perceived harm of e-liquids were analyzed using logistic and multinomial regressions adjusted for age, sex, race or ethnicity, country, vaping, and smoking status.

**Results:**

Compared with branded e-liquid packs, more youth reported no interest in trying e-liquids in white (aOR = 1.48, 95% CI = 1.34 to 1.64) or olive (aOR = 1.62, 95% CI: 1.47 to 1.80) standardized packs. Compared with branded e-liquid packs, more youth inaccurately perceived e-liquids in white (aOR = 1.22, 95% CI: 1.11 to 1.34) or olive (aOR = 1.29, 95% CI: 1.18 to 1.41) standardized packs as equally or more harmful than smoking. E-liquid nicotine levels displayed on packs were not associated with youth interest in trying or harm perceptions of using e-liquids.

**Conclusions:**

Among 16- to 19-year-old youth from England, Canada, and the United States, standardized packaging of e-liquids was associated with lower interest in trying and higher health risk perceptions.

**Implications:**

Branded packaging of vaping products appeal to youth and might prompt nicotine use among those who had never smoked. This study suggests that restricting branding elements on e-liquid packaging is associated with youth's lower interest in trying e-liquids and higher misperceptions that vaping is equally or more harmful than smoking. Standardized packaging might reduce appeal of vaping among youth, but its potential to discourage vaping for harm reduction should also be considered.

## Introduction

Vaping is substantially less harmful than smoking,^1^ and marketing of vaping products could facilitate harm reduction if it prompts switching completely from smoking cigarettes to vaping. However, current state of vaping promotions may encourage vaping among those who have never smoked. For instance, youth (15- to 24-year-olds) perceive that vaping marketing is directed towards young people^2^ and nonsmokers,^3^ which is supported by evidence that promotion of vaping products appeals more to youth than to adults who smoke.^3,4^ Furthermore, vaping offers a milder use experience than smoking, which increases appeal among women, young people and those inexperienced with smoking,^5^ and vaping products are sold in various flavors promoted in colorful packaging that attracts youth attention.

Packaging is important for promoting tobacco and vaping products.^6,7^ Packaging of vaping products—devices, pods, and e-liquids—often contains elements that may appeal to youth; for instance, packaging often emphasizes sweet or fruit flavors,^8^ includes cartoons,^9^ or is designed to resemble food or drink products that are mostly marketed to youth, such as candy or soda.^10^ To mitigate such promotion, standardized (ie, plain) packaging of vaping products in a dark olive color and with mandatory health warnings has been adopted in Israel and the Netherlands.^7^ Although no studies have yet assessed the effect of standardized vaping product packaging in practice, experimental data suggest that it might moderate the appeal of vaping products among youth. For example, recent findings showed that standardized packaging reduced the appeal of vaping products among youth aged 16 to 19 in England, Canada, and the United States,^11^ and online experiments in Great Britain found that standardized olive packaging for vaping products reduced the appeal of vaping among youth aged 11 to 18 while having little impact on interest in vaping for smoking cessation among adults who smoked.^12^ However, no studies have assessed how youth perceive standardized packs of e-liquids that are used with refillable devices popular among youth who vape.^13^ The abundance of flavors and types of e-liquids requires researching how their packaging is associated with youth interest in trying vaping.

Perceptions of vaping harms health can influence people’s vaping and smoking behavior.^1^ To date, studies of standardized packs for vaping products have used the dark olive color mandated for standardized cigarette packs in certain countries, including England and Canada.^7^ Tobacco and nicotine products in lighter-colored packs might be perceived as less harmful than those in darker-colored packs,^14^ and lighter-colored packaging might help distinguish vaping products from more harmful tobacco cigarettes in standardized olive packs. To assess whether the color of standardized e-liquid packs is associated with youth interest in trying e-liquids and harm perceptions, our study compares branded, standardized white, and standardized olive packs of e-liquids.

Youth and adults often inaccurately attribute most of the health risks of smoking to nicotine,^15,16^ therefore nicotine content of e-liquids could influence their appeal and perceptions of risk. However, many people who vape have lately transitioned towards using salt-based, higher nicotine concentration e-liquids,^13^ and evidence suggests that people rarely focus on the information on nicotine content of vaping products.^17^ Similarly, substantial proportions of youth report not knowing the nicotine content of their vaping products,^13^ do not understand how to interpret information on nicotine strength,^18^ and tend to disregard health warnings about nicotine addiction on packaging of vaping products.^19^ To explore youth perceptions of e-liquid nicotine content, our study compares how different nicotine levels displayed on e-liquid packs are associated with youth interest in trying and harm perceptions of the e-liquids.

Youth samples from England, Canada, and the United States were recruited online to an experiment exploring how branded, standardized olive, and standardized white e-liquid packaging with low or high e-liquid nicotine content displayed on the packs are associated with youth (1) interest in trying e-liquids and (2) perceptions of health harms of using e-liquids.

## Methods

The analysis plan including descriptions of measures was preregistered on the Open Science Framework (https://osf.io/jbv6u).^20^

### Study Design and Sample

Study data were from wave 5 (August–September 2021) of the International Tobacco Control Policy Evaluation Project Youth Tobacco and Vaping Survey (ITC-Y), a repeated cross-sectional online survey of youth aged 16 to 19 in England, Canada, and the United States. Respondents were recruited through the Nielsen Consumer Insights Global Panel, directly or through their parents. Participants received remuneration according to their panel’s incentive structure. The survey received ethics clearance through the University of Waterloo Research Ethics Committee (ORE#21847/31017) and King’s College London Psychiatry, Nursing and Midwifery Research Ethics Subcommittee. Further details can be found online.^21^

Overall, 13 801 respondents were included in the e-liquid packaging experiment. Participants were excluded if they answered “don’t know” or “refused” to questions about their race or ethnicity (*n* = 247), vaping or smoking status (*n* = 30), and to the e-liquid selection (*n* = 108) or perceived harm (*n* = 46) questions that served as outcome variables. Using a 3 × 2 (three packaging and two nicotine content conditions) between-subject experimental design, participants were randomized to one of six conditions: (1) branded pack and low-nicotine e-liquid, (2) branded pack and high-nicotine e-liquid; (3) standardized white pack and low-nicotine e-liquid (4) standardized white pack and high-nicotine e-liquid, (5) standardized olive pack and low-nicotine e-liquid and (6) standardized olive pack and high-nicotine e-liquid.

**Figure 1.**
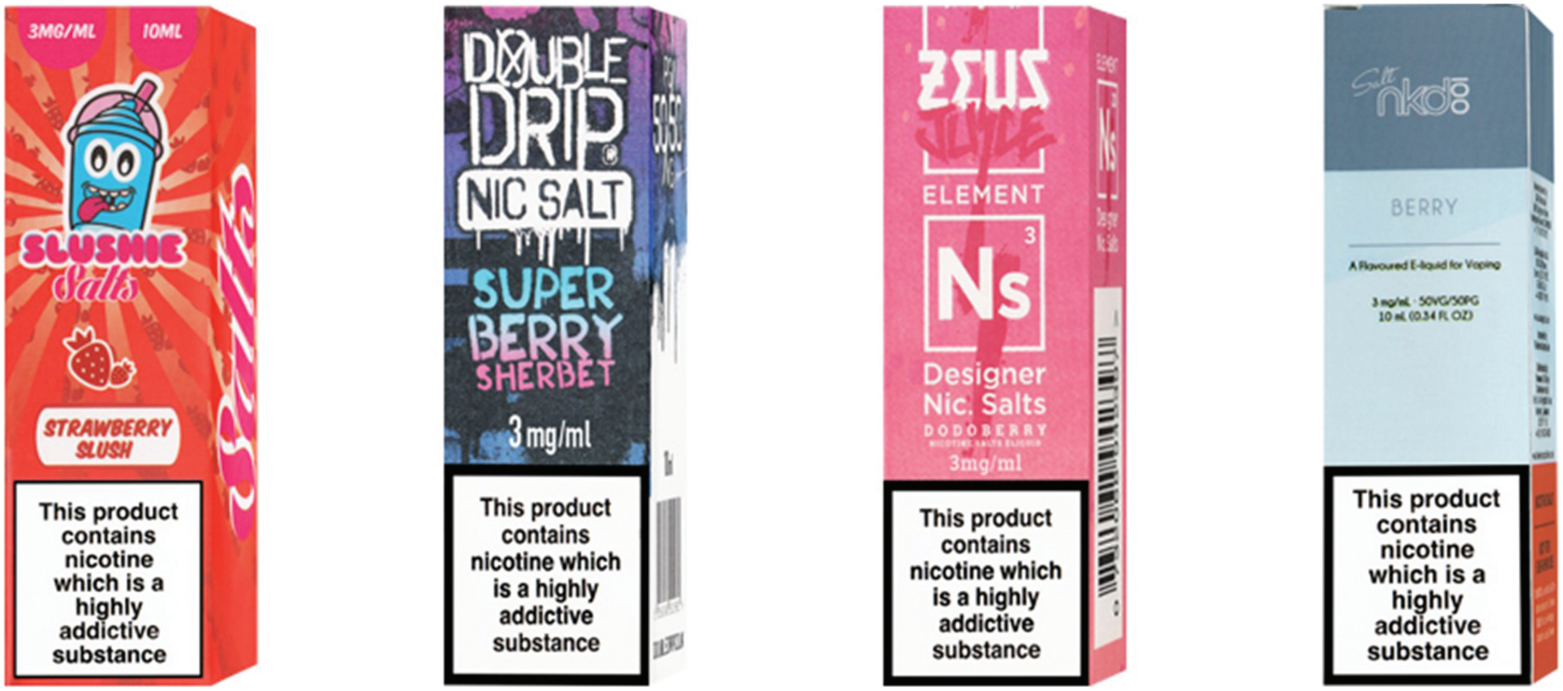
Branded packs of low (3 mg per mL) nicotine e-liquid (condition 1) for participants in England. From left to right: youth-oriented, male-oriented, female-oriented, and neutral designs.

Within each condition, participants viewed images of four brands of e-liquid packs, with country-specific health warnings (England: “This product contains nicotine which is a highly addictive substance”^22^; Canada: “WARNING: Nicotine is highly addictive. Health Canada AVERTISSEMENT: La nicotine crée une forte dépendance. Santé Canada”^23^; United States: “WARNING: This product contains nicotine. Nicotine is an addictive chemical.”^24^) and nicotine content descriptors reflecting local regulations (3 mg per mL and 20 mg per mL nicotine in England and Canada; 3 mg per mL and 59 mg per mL, the nicotine level of the most popular pod vaping product at the time of the study,^13^ in the United States). Pack designs for the branded packaging condition represented the range on the market in the three countries, including youth-oriented, male-oriented, female-oriented, or neutral (Figure 1). All e-liquids were berry-flavored because fruit flavors were the most popular among youth at the time of the survey.^25^ All variations of e-liquid packs can be found in the preregistration document (https://osf.io/jbv6u).^20^

### Measures

#### Primary Outcome Variables

*Interest in Trying E-liquids*. Participants were shown five evenly sized images based on the condition they had been assigned to—four images of e-liquid packs (youth-oriented, male-oriented, female-oriented, neutral) presented in random order and a text box stating “I have no interest in trying any of these products”—and were asked, “Which of the following e-liquids would you be most interested in trying?.” They could select one of the four e-liquid packs displayed, “I have no interest in trying any of these products,” “Don’t know” or “Refused.” Responses were categorized as “No interest in trying any of the e-liquid products” and “Interest in trying any of the shown e-liquids or don’t know” (reference category). Participants who selected “Refused” were excluded from data analyses.

*Perceived Harm of E-liquids*. Participants were then shown an image of the youth-oriented e-liquid pack based on the condition they had been assigned to and were asked “How harmful do you think it is to vape this product?.” Response options included “Not at all harmful,” “Harmful, but less harmful than smoking cigarettes,” “As harmful as smoking cigarettes,” “More harmful than smoking cigarettes,” “Don’t know” or “Refused.” Responses were categorized as “Not at all harmful,” “Less harmful than smoking” (the correct response^1^ and reference category), or “As harmful or more harmful than smoking or don’t know.” Participants who selected “Refused” were excluded from data analyses.

#### Intervention Variables

E-liquid packaging condition (branded, standardized white, standardized olive; pairwise comparisons) and e-liquid nicotine condition (low-nicotine: 3 mg per mL, as reference category; high-nicotine: 20 mg per mL or 59 mg per mL).

#### Sociodemographic Variables

Country (England, Canada, the United States; pairwise comparisons), age (16 as reference category, 17, 18, 19), sex (female as reference category, male), and race or ethnicity (a derived variable for three countries; white as reference category, any other). Participants who answered “Don’t know” or “Refused” about sex or race or ethnicity were excluded from all data analyses.

#### Vaping and Smoking Variables

Vaping status (never vaped; former or experimental vaping (ie, ever vaped but not in the past 30 days); vaped in past 30 days), smoking status (never smoked; former or experimental smoking (ie, ever smoked but not in the past 30 days); smoked in past 30 days) and a combined vaping and smoking status variable with five mutually exclusive categories were created (never used (ie, never vaped and never smoked); former vaping or smoking (ie, ever vaped or ever smoked, but neither in the past 30 days); vaped in past 30 days (ie, vaped but did not smoke in the past 30 days); smoked in past 30 days (ie, smoked but did not vape in the past 30 days); vaped and smoked in past 30 days).

### Data Analyses

Frequencies were calculated for sociodemographic and vaping and smoking characteristics in total, by e-liquid packaging condition, and by perceived harm of e-liquids. Pearson’s *χ2* tests and Cramer’s V (*φ*_*c*_—a measure of the strength of association between two categorical variables) were used to test participants’ randomization to experimental conditions by country, sex, age, race or ethnicity, and vaping and smoking status.

To examine youth interest in trying e-liquids by packaging condition (aim 1), a logistic regression model with “No interest in trying e-liquid products” as the outcome was regressed onto sociodemographic, vaping and smoking, e-liquid packaging, and nicotine condition variables, followed by an interaction between e-liquid packaging and nicotine conditions. The interaction term was excluded from the final model if it had not statistically significantly improved the model fit (defined by *χ2* differences between models with and without the interaction).

To examine youth harm perceptions by packaging condition (aim 2), a multinomial logistic regression model with e-liquids’ perceived harm as an outcome variable was regressed onto sociodemographic, vaping and smoking, e-liquid packaging, and nicotine condition variables, followed by an interaction between e-liquid packaging and nicotine conditions. The interaction term was excluded from the final model if it had not significantly improved the model fit (defined by *χ2* differences between models with and without the interaction).

In the preregistered analysis (https://osf.io/jbv6u)^20^ we planned to include vaping and smoking variables as independent predictors, but a combined vaping and smoking status variable was used instead to account for participants’ concurrent vaping and smoking. To test how this change affected the logistic regression results for youth interest in trying e-liquids, an additional regression model with independent vaping and smoking predictors was estimated. To test whether youth interest in trying e-liquids and harm perceptions differed by vaping and smoking status, additional analyses have been conducted including interaction terms between participants’ vaping and smoking status (“past 30-day vaping” as reference category) and packaging condition (“branded” as reference category) to the final logistic and multinomial regressions.

## Results

Table 1 provides sample characteristics by e-liquid packaging condition.

**Table 1.** Sample Characteristics by E-liquid Packaging Condition, 2021 ITC Youth Survey (*n* = 13 801)

	Total, % (*n*)	Branded, % (*n*)	Standardized white, % (*n*)	Standardized olive, % (*n*)
Total	100.0 (13801)	33.3 (4600)	33.4 (4606)	33.3 (4595)
*Nicotine condition* ^†^				
Low-nicotine (3mg per mL)	49.9 (6892)	49.9 (2297)	50.0 (2304)	49.9 (2291)
High-nicotine (20 or 59mg per mL)	50.1 (6909)	50.1 (2303)	50.0 (2302)	50.1 (2304)
*Test statistics*		*χ* ^*2*^(2) = 0.02, *p* = .99, φ_*c *_= .001		
*Sex*				
Female	68.8 (9499)	68.4 (3145)	68.8 (3167)	69.4 (3187)
Male	31.2 (4302)	31.6 (1455)	31.2 (1439)	30.6 (1408)
*Test statistics*		*χ* ^*2*^(2) = 1.1, *p* = .59, φ_*c *_= .01		
*Age (years)*				
16	19.4 (2681)	19.8 (909)	19.7 (909)	18.8 (863)
17	23.8 (3282)	23.6 (1084)	24.0 (1105)	23.8 (1093)
18	33.0 (4556)	33.5 (1539)	32.0 (1475)	33.6 (1542)
19	23.8 (3282)	23.2 (1068)	24.3 (1117)	23.9 (1097)
*Test statistics*		*χ* ^*2*^(6) = 4.8, *p* = .57, φ_*c *_= 0.01		
*Race or ethnicity*				
White	55.0 (7589)	55.5 (2552)	54.6 (2513)	54.9 (2524)
Any other	43.2 (5965)	42.6 (1959)	43.6 (2010)	43.4 (1996)
*Test statistics*		*χ* ^*2*^(2) = 1.0, *p* = .61, φ_*c *_= .01		
Don’t know or refused	1.8 (247)	1.9 (89)	1.8 (83)	1.6 (75)
*Country*				
Canada	33.4 (4604)	33.5 (1539)	33.3 (1532)	33.4 (1533)
England	31.3 (4316)	31.7 (1460)	31.3 (1441)	30.8 (1415)
US	35.4 (4881)	34.8 (1601)	35.5 (1633)	35.8 (1647)
*Test statistics*		*χ* ^*2*^(4) = 1.4, *p* = .84, φ_*c *_= .01		
*Smoking status*				
Smoked in past 30 days	4.5 (613)	4.4 (204)	4.7 (217)	4.2 (192)
Former or experimental smoking	28.4 (3915)	27.5 (1261)	29.4 (1352)	28.4 (1302)
Never smoked	67.1 (9243)	68.1 (3123)	65.9 (3027)	67.4 (3093)
*Test statistics*		*χ* ^*2*^(4) = 1.4, *p* = .84, φ_*c *_= .01		
Missing	0.2 (30)	0.1 (12)	0.1 (10)	0.1 (8)
*Vaping status*				
Vaped in past 30 days	16.8 (2319)	16.6 (762)	16.8 (774)	17.0 (783)
Former or experimental vaping	23.7 (3277)	23.5 (1081)	24.1 (1109)	23.7 (1087)
Never vaped	60.1 (8205)	59.9 (2757)	59.1 (2723)	59.3 (2725)
*Test statistics*		*χ* ^*2*^(4) = 2.3, *p* = .68, φ_*c *_= .01		
*Vaping and smoking status*				
Never used	52.4 (7230)	52.8 (2429)	51.4 (2369)	52.9 (2432)
Former vaping or smoking	28.8 (3977)	28.5 (1312)	29.5 (1361)	28.4 (1304)
Vaped in past 30 days	14.1 (1951)	14.0 (643)	14.1 (649)	14.3 (659)
Vaped and smoked in past 30 days	2.5 (351)	2.4 (112)	2.6 (121)	2.6 (118)
Smoked in past 30 days smoker	1.9 (262)	2.0 (92)	2.1 (96)	1.6 (74)
*Test statistics*		*χ* ^*2*^(8) = 6.2, *p* = .63, φ_*c *_= .01		
Missing	0.2 (30)	0.3 (12)	0.2 (10)	0.2 (8)

^†^The experiment included 6 conditions based on the interaction between e-liquid packaging type and nicotine level conditions.

The study sample included more females (68.8%) than males (31.2%), one-third were 18 years old (33.0%) and 55.0% identified as white race or ethnicity. More than half had never tried vaping or smoking (52.4%), 28.8% had formerly vaped or smoked, 14.1% had only vaped in the past 30 days, 2.5% had both vaped and smoked in the past 30 days, and 1.9% had only smoked in the past 30 days. Participants’ characteristics did not differ statistically significantly by e-liquid packaging conditions, indicating that randomization was successful (Table 1).

### No Interest in Trying E-liquid Products

Participants who refused to answer the e-liquid selection question (*n* = 108, 0.8% of the sample) were excluded from the analysis of interest in trying e-liquids by packaging condition. Table 2 shows logistic regression findings for the “No interest in trying e-liquid products” outcome.

**Table 2.** Logistic Regression Model for Selecting “I Have No Interest in Trying any of These Products,” Adjusted for Sociodemographic, Vaping and Smoking, and Intervention Variables (*n* = 13 426).

	% (*n*)	aOR (95% CI)	*p* value
Intercept		**11.2 (9.53 to 13.20)**	**<.001**
Packaging condition			
Branded	65.0 (2912)	Ref	
Standardized white	71.0 (3171)	**1.48 (1.34 to 1.64)**	**<.001**
Standardized olive	72.9 (3266)	**1.62 (1.47 to 1.80)**	**<.001**
*Nicotine condition*			
Low (3 mg per mL)	69.8 (4686)	Ref	
High (20 or 59 mg per mL)	69.5 (4663)	0.95 (0.87 to 1.03)	.24
*Sex*			
Female	68.8 (6374)	Ref	
Male	71.4 (2975)	**1.15 (1.04 to 1.26)**	**.004**
*Age (years)*			
16	76.1 (1976)	Ref	
17	74.0 (2361)	1.01 (0.88 to 1.15)	.92
18	66.6 (2960)	**0.81 (0.71 to 0.91)**	**<.001**
19	64.1 (2052)	**0.79 (0.69 to 0.90)**	**<.001**
*ace or ethnicity*			
White	68.3 (5142)	Ref	
Any other	71.3 (4207)	**0.83 (0.76 to 0.91)**	**<.001**
*Country*			
England	65.1 (2740)	Ref	
Canada	72.7 (3234)	**1.43 (1.29 to 1.59)**	**<.001**
US	70.7 (3375)	**1.22 (1.10 to 1.36)**	**<.001**
*Vaping and smoking status*			
Never used	87.3 (6116)	Ref	
Former vaping or smoking	62.4 (2437)	**0.24 (0.22 to 0.27)**	**<.001**
Vaped in past 30 days	30.9 (592)	**0.06 (0.06 to 0.07)**	**<.001**
Vaped and smoked in past 30 days	22.4 (77)	**0.04 (0.03 to 0.05)**	**<.001**
Smoked in past 30 days	49.2 (127)	**0.14 (0.11 to 0.19)**	**<.001**
Packaging * Nicotine condition ^†^			*χ* ^*2*^(2) = 0.06, *p* = .97

^†^The interaction term between packaging and nicotine conditions was not statistically significant and was removed from the final model.

#### No Interest by E-liquid Packaging Conditions

Compared with the branded e-liquid packs, participants were statistically significantly more likely to report no interest in trying e-liquids in the standardized white or the standardized olive packs (Table 2). Participants reported a similar lack of interest in trying e-liquids in the standardized white and the standardized olive packs (aOR = 1.09, 95% CI: 0.99 to 1.22, *p* = .091).

#### No Interest in E-liquid Nicotine Conditions

Nicotine level descriptors were not statistically significantly associated with participants reporting no interest in trying e-liquids and there was no interaction between nicotine and packaging conditions (Table 2).

#### Other Covariates and Additional Analyses

Participants who were younger than 18 years, male, and identified as white race or ethnicity were statistically significantly more likely to report no interest in trying e-liquids (Table 2). Participants from Canada and the United States were more likely to report no interest in trying e-liquids than participants from England (Table 2); participants from Canada were more likely to report no interest in trying e-liquids than participants from the United States (aOR = 1.16, 95% CI: 1.05 to 1.30, *p* = .003). Youth who had formerly vaped or smoked were over 4 times less likely, those who smoked in the past 30 days over 7 times less likely, those who vaped in the past 30 days over 16 times less likely, and those who vaped and smoked in the past 30 days around 25 times less likely to report no interest in trying e-liquids compared with youth who had never smoked or vaped (Table 2).

Findings did not differ when vaping and smoking variables were included independently in the regression model (Supplementary Table 1). Youth interest in trying e-liquids did not differ by vaping and smoking status, as the interaction between vaping and smoking status and packaging condition was not statistically significant (*χ*^*2*^(8)=7.3, *p*=0.50). “Don’t know” responses were uncommon (2.5%), and the interpretation of findings did not differ when analyzing “Don’t know” responses separately (Supplementary Table 2) or in combination with the “Interest in trying any of the shown e-liquids” response (Table 2).

### Perceived Harm of E-liquid Products

Forty-six (0.3% of the sample) participants who refused to answer the perceived harm question were excluded from the analysis of harm perceptions. Supplementary Table 3 provides sample characteristics by participants’ perceived harm of e-liquids, including “don’t know” and “refused” responses. More than half of youth (53.8%) perceived vaping the e-liquid shown either as harmful (43.2%) or more harmful than smoking cigarettes (10.6%), 31.6% perceived it as harmful but less harmful than smoking, 12.4% did not know and 2.1% perceived vaping the e-liquid shown as not at all harmful.

Table 3 shows the results of a multinomial regression predicting “not at all harmful” and “as harmful or more harmful than smoking or don’t know” responses in contrast with the correct response that vaping the e-liquid shown is “less harmful than smoking.”

**Table 3.** Multinomial Logistic Regression Model Predicting “Not at all Harmful” and “As Harmful or More Harmful or Don’t Know” Responses Versus “Less Harmful Than Smoking” Response as a Reference, Adjusted for Sociodemographic, Vaping and Smoking, and Intervention Variables (*n* = 13 485).

	Less harmful than smoking (reference category)	Not at all harmful			As harmful, more harmful or don’t know		
	% (*n*)	% (*n*)	aOR (95% CI)	*p* value	% (n)	aOR (95% CI)	*p* value
Intercept			**0.04 (0.02 to 0.06)**	**<.001**		**2.73 (2.38 to 3.13)**	**<.001**
*Packaging condition*							
Branded	34.6 (1554)	2.7 (123)	Ref		62.6 (2810)	Ref	
Standardized white	31.1 (1399)	1.6 (70)	**0.62 (0.46 to 0.84)**	**.002**	67.3 (3024)	**1.22 (1.11 to 1.34)**	**<.001**
Standardized olive	29.8 (1343)	1.8 (81)	**0.75 (0.56 to 0.998)**	**.049**	68.4 (3081)	**1.29 (1.18 to 1.41)**	**<.001**
*Nicotine condition*							
Low (3 mg per mL)	32.7 (2200)	2.2 (151)	Ref		65.1 (4381)	Ref	
High (20 or 59 mg per mL)	31.0 (2096)	1.8 (123)	0.87 (0.68 to 1.11)	.26	67.1 (4534)	1.07 (0.997 to 1.16)	.062
*Sex*							
Male	33.8 (1415)	2.8 (119)	Ref		63.4 (2655)	Ref	
Female	31.0 (2881)	1.7 (155)	**0.63 (0.49 to 0.81)**	**<.001**	67.3 (6260)	**1.13 (1.05 to 1.23)**	**.002**
*Age (years)*							
16	28.9 (757)	1.8 (47)	Ref		69.3 (1811)	Ref	
17	30.6 (982)	2.1 (66)	1.06 (0.72 to 1.57)	.77	67.3 (2158)	0.94 (0.84 to 1.06)	.30
18	33.2 (1479)	2.2 (99)	1.00 (0.69 to 1.44)	.99	64.6 (2880)	**0.88 (0.79 to 0.98)**	**.020**
19	33.6 (1078)	1.9 (62)	0.80 (0.53 to 1.19)	.26	64.4 (2066)	**0.86 (0.76 to 0.97)**	**.011**
*Race or ethnicity*							
White	34.5 (2603)	2.2 (164)	Ref		63.3 (4780)	Ref	
Any other	28.5 (1693)	1.9 (110)	1.22 (0.95 to 1.58)	.126	69.6 (4135)	1.08 (0.995 to 1.16)	.066
*Country*							
England	43.1 (1826)	2.6 (109)	Ref		54.3 (2299)	Ref	
Canada	28.6 (1276)	1.8 (79)	1.02 (0.76 to 1.39)	.88	69.6 (3104)	**1.93 (1.76 to 2.12)**	**<.001**
US	24.9 (1194)	1.8 (86)	1.27 (0.94 to 1.71)	.124	73.3 (3512)	**2.28 (2.07 to 2.50)**	**<.001**
*Vaping and smoking status*							
Never used	25.3 (1787)	1.0 (67)	Ref		73.7 (5198)	Ref	
Former vaping or smoking	34.2 (1338)	1.7 (68)	1.41 (0.999 to 2.00)	.051	64.1 (2511)	**0.67 (0.62 to 0.73)**	**<.001**
Vaped in past 30 days	48.0 (922)	5.4 (104)	**3.24 (2.35 to 4.47)**	**<.001**	46.5 (893)	**0.33 (0.30 to 0.37)**	**<.001**
Vaped and smoked in past 30 days	50.1 (170)	8.0 (27)	**4.67 (2.87 to 7.60)**	**<0.001**	41.9 (142)	**0.34 (0.27 to 0.43)**	**<.001**
Smoked in past 30 days	30.6 (79)	3.1 (8)	**2.94 (1.35 to 6.39)**	**.007**	66.3 (171)	0.98 (0.74 to 1.29)	.87
Packaging * Nicotine condition ^†^							*χ* ^*2*^(4) = 2.3, *p* = .68

^†^The interaction term between packaging and nicotine conditions was not statistically significant and was removed from the final model.

Cells in bold indicate statistically significant differences compared with the reference category.

#### Perceived Harm by E-liquid Packaging Conditions

Compared with the “less harmful than smoking” response, participants were statistically significantly more likely to perceive vaping e-liquids as “as harmful or more harmful than smoking or don’t know” in standardized white packs and standardized olive packs than e-liquids in branded packs (Table 3). The proportions of “don’t know” responses were very similar between the three packaging conditions, suggesting that the difference was because of fewer participants reported that vaping the e-liquid in a branded pack (50.5%) was “as harmful or more harmful” than smoking compared with the standardized white (55.5%) and standardized olive (55.3%) packs (Supplement Table 3).

Compared with the “less harmful than smoking” response, vaping e-liquids in standardized white and standardized olive packs were less likely to be perceived as “not at all harmful” than vaping e-liquids in branded packs (Table 3).

Participants’ relative harm perceptions of the e-liquids in standardized white and standardized olive packs did not differ statistically significantly when comparing responses “not at all harmful” (1.6% vs. 1.8%; aOR = 1.20, 95% CI: 0.86 to 1.67, *p* = .28) and “as harmful or more harmful than smoking or don’t know” (67.3% vs. 68.4%; aOR = 1.06, 95% CI: 0.96 to 1.16, *p* = .26) with the correct response that vaping e-liquid shown was “less harmful than smoking.”

#### Perceived Harm by E-liquid Nicotine Conditions

Participants’ relative harm perceptions of e-liquids with low—versus high-nicotine levels did not differ statistically significantly (Table 3). The interaction between nicotine and packaging conditions was not statistically significant (*χ*^*2*^(4) = 2.3, *p* = .68).

#### Other Covariates and Additional Analyses

Compared with the “less harmful than smoking” response, females perceived e-liquids less likely as “not at all harmful” and more likely as “as harmful or more harmful than smoking or don’t know” than males (Table 3). Compared with 16-year-olds, 18- or 19-year-olds were less likely to perceive e-liquids shown as “as harmful or more harmful than smoking or don’t know” than “less harmful than smoking” (Table 3). Participants from Canada and the United States were more likely to perceive e-liquids shown “as harmful or more harmful than smoking or don’t know” than participants from England (Table 3). Participants from the United States were also more likely to perceive e-liquids shown as “as harmful or more harmful than smoking or don’t know” than participants from Canada (aOR = 1.18, 95% CI: 1.07 to 1.30, *p* < .001).

Respondents who had both vaped and smoked in the past 30 days, or only vaped in the past 30 days, most often selected the “less harmful than smoking” option when assessing the harm of vaping e-liquids (Table 3). In terms of perceiving the e-liquids shown as “as harmful or more harmful than smoking or don’t know,” only respondents who smoked in the past 30 days did not show statistically significant difference compared with respondents who had never vaped or smoked (Table 3).

#### Interaction Between Vaping and Smoking Status and E-liquid Packaging Condition

The interaction between vaping and smoking status and packaging condition was statistically significant (*χ*^*2*^(16) = 31.1, *p* = .013), suggesting that some vaping and smoking status groups perceived harm of e-liquids differently based on their packaging. Specifically, those who had never vaped or smoked, and those who formerly vaped or smoked were more likely to perceive e-liquids in standardized white and standardized olive packs as “as harmful, more harmful or don’t know” than e-liquids in branded packs, in contrast with those who had vaped in the past 30 days (Supplementary Table 4). Harm perceptions of those who had both vaped and smoked in the past 30 days, or only smoked in the past 30 days, did not differ between packaging designs, when compared with harm perceptions of those who had vaped in the past 30 days. Harm perceptions by vaping and smoking status and packaging conditions are presented in Supplementary figure 1.

## Discussion

Among youth aged 16 to 19 from England, Canada, and the United States, more reported no interest in trying e-liquids in white or olive standardized packs than in branded packs. Compared with branded packs, vaping e-liquids in white and olive standardized packs were less likely perceived by youth as not at all harmful and more likely perceived as equally or more harmful than smoking (or don’t know). Neither interest in trying nor perceived harm of e-liquids differed between standardized packs in olive or white, or between packs with low or high-nicotine levels displayed. To the best of our knowledge, this is the first study focusing on standardized packaging of e-liquids and their harm perceptions, and its results extend earlier findings on e-cigarette devices^11,12^ by demonstrating that standardized packaging of e-liquids reduces youth interest in trying these products but could also increase inaccurate harm perceptions of e-liquids.

Olive-colored packaging might be associated with cigarette smoking and its health harm, but our study did not find differences in youth interest in trying or harm perceptions of e-liquids in white or olive standardized packs. E-liquids in standardized packs of either color were associated with significantly lower youth interest in trying than e-liquids in branded packs, implying that the design elements of branded e-liquid packaging are associated with youth interest in trying these products.

While fewer participants perceived e-liquids in standardized white or standardized olive packs as not at all harmful than e-liquids in branded packs, in general, we found a stronger association between standardized packaging and greater misperceptions that using e-liquids was equally or more harmful than smoking. These misperceptions were most pronounced among those who had never vaped or smoked, those who formerly vaped or smoked, and those who had smoked in the past 30 days. Additional analysis found that those who had never vaped or smoked and those who formerly vaped or smoked were more likely to perceive e-liquids in standardized than branded packs as equally or more harmful than smoking, while e-liquid harm perceptions among those who had vaped or smoked in the past 30 days did not differ between packaging conditions. Relative harm perceptions are associated with vaping and smoking behaviors,^1^ and our results show that misperceptions about e-liquid relative harm might deter youth from trying vaping. The potential effects of standardized e-liquid packaging on vaping for harm reduction among adults who smoke need further exploration. Recent findings in Great Britain found a greater impact of standardized e-cigarette packaging in reducing the appeal of vaping among youth, with little impact on interest in vaping for smoking cessation among adults who smoked.^12^

There was little evidence to suggest that the nicotine level displayed on e-liquid packs was associated with youth interest in trying e-liquids or perceptions of harm. Prior research showed that most youth inaccurately attribute health harms of smoking to nicotine,^16^ but youth in our study did not perceive high-nicotine e-liquids as more harmful. One explanation for this might be a relatively low salience of the nicotine level descriptor on e-liquid packs, which might have been overlooked by participants. Alternatively, youth might underestimate the strength of nicotine concentrations in e-liquids,^18^ or nicotine levels might not be important for youth interest in trying or harm perceptions of e-liquids, consistent with findings that youth disregard the seriousness of nicotine addiction,^13,19,26^ and that noticing nicotine warnings on vaping products may not be associated with harm perceptions or intentions to vape.^27,28^

As expected, youth who reported vaping were more likely to correctly perceive e-liquids as less harmful than smoking. Youth who reported smoking, however, were more likely to incorrectly report that vaping e-liquids were equally or more harmful than smoking. The misperceptions may stem from unrealistic optimism about smoking risks among youth who smoke,^29^ thereby equating harms of vaping to harms of smoking. While incorrect vaping harm perceptions among adults who smoke might facilitate continued smoking and deter them from vaping for harm reduction,^30^ our findings indicate that the same might be true for youth.

### Implications and Future Research

Public health policy in some countries aims to strike a balance between informing people that vaping is not risk-free and encouraging people who smoke to switch completely from smoking to vaping to reduce harm to health. Our findings show that standardized e-liquid packaging might reduce both youth interest in trying e-liquids and perceptions that vaping e-liquids is not at all harmful, but it may also increase misperceptions about the relative harms of vaping. For example, youth who never or formerly vaped or smoked were more likely to misperceive the e-liquids displayed in standardized white or olive packs as equally or more harmful than smoking compared with branded packs. While perceiving vaping to be as harmful as smoking might deter youth from starting vaping,^1^ future study could explore whether the misperceptions equating vaping and smoking in terms of health harms also prevent youth from starting smoking.

The extent to which standardized packaging of vaping products could deter those who smoke from switching to vaping is another important question. Our study shows that around two-thirds of youth who smoke misperceived that the e-liquids displayed were equally or more harmful than cigarette smoking, regardless of the packaging condition. While preliminary evidence suggests that restricting branding elements on vaping product packaging has little impact on interest in vaping to stop smoking among adults who smoke,^12^ future research needs to clarify how standardizing vaping product packaging might affect vaping for harm reduction and smoking cessation. Also, the study examined packaging for e-liquids to be used in refillable vaping products; future studies should examine the impact of standardized packaging on disposable and cartridge or pod-based e-cigarettes that are most popular among youth.^25^ Finally, the feasibility of introducing standardized packaging for vaping products is unclear in the countries studied. A form of standardized packaging for vaping products was implemented^7^ and later repealed in the Canadian province of British Columbia, and England is currently consulting on ways to reduce vaping among youth,^31^ where standardized packaging could be one of the options. In the United States, however, standardized packaging for cigarettes or other tobacco products has not been implemented, so standardized packaging for vaping products seems unlikely.

### Limitations and Strengths

In this experiment, youth answered questions about e-liquid images viewed on screen and not on actual e-liquid packs, which may limit the validity of research findings. Also, the e-liquid nicotine conditions did not include a nicotine-free option and participants were not asked if they had noticed the nicotine level descriptors, thus limiting our conclusions about youth perceptions of nicotine in e-liquids. The sample was recruited from commercial research panels, so findings might not be representative of youth within the three countries. Nevertheless, this was the first randomized experiment of how a large sample of 16- to 19-year-olds across three countries perceives e-liquids in standardized and branded packs, in terms of interest in trying and harm to health.

## Conclusions

Among 16- to 19-year-old youth from England, Canada, and the United States, standardized e-liquid packaging was associated with lower interest in trying e-liquids and higher perceptions of the harms of vaping, including compared to smoking. Nicotine levels displayed on e-liquid packs were not associated with youth interest in trying or with harm perceptions. Future research should examine how standardized packaging of different vaping products might effectively reduce the appeal of vaping among youth without discouraging switching to vaping among adults who smoke.

## Supplementary Material

A Contributorship Form detailing each author’s specific involvement with this content, as well as any supplementary data, are available online at https://academic.oup.com/ntr.

ntad144_suppl_Supplementary_Data

## Data Availability

The manuscript describes analyses of secondary data. The code is available online at https://osf.io/34ncz/files/osfstorage/6492b55638091104853c313d.
