# Supplementary material for: Impact of E-liquid Packaging on Vaping Product Perceptions Among Youth in England, Canada, and the United States: A Randomized Online Experiment
Source: Nicotine Tob Res. 2023 Aug 5;26(3):370–9. doi: 10.1093/ntr/ntad144 (PMC10882429; doi:10.1093/ntr/ntad144)
Supplement: ntad144_suppl_Supplementary_Data [file ntad144_suppl_supplementary_data.docx]

**Impact of e-liquid packaging on vaping product perceptions among youth in England, Canada, and the United States; a randomised online experiment**

# Supplement

Supplement table 1 Logistic regression model for selecting ‘I have no interest in trying any of these products’ adjusted for sociodemographic, vaping, smoking and intervention variables (n=13275)*.

|  | **% (n)** | **aOR (95% CI)** | **p value** |
| --- | --- | --- | --- |
| **Intercept** |  | **4.86 (4.18-5.66)** | **<0.001** |
| **Packaging condition** |  |  |  |
| Branded | 65.2 (2888) | Ref |  |
| Standardised white | 71.2 (3149) | **1.50 (1.36-1.67)** | **<0.001** |
| Standardised olive | 73.3 (3243) | **1.65 (1.49-1.83)** | **<0.001** |
| **Nicotine condition** |  |  |  |
| Low (3 mg/mL) | 70.2 (4649) | Ref |  |
| High (20 or 59 mg/mL) | 69.6 (4631) | 0.95 (0.87-1.03) | 0.22 |
| **Sex** |  |  |  |
| Female | 69.2 (6324) | Ref |  |
| Male | 71.6 (2956) | **1.15 (1.05-1.26)** | **0.003** |
| **Age (years)** |  |  |  |
| 16 | 76.3 (1960) | Ref |  |
| 17 | 74.5 (2345) | 1.04 (0.91-1.19) | 0.58 |
| 18 | 66.8 (2939) | **0.87 (0.76-0.98)** | **0.023** |
| 19 | 64.4 (2036) | **0.86 (0.76-0.99)** | **0.029** |
| **Race/ethnicity** |  |  |  |
| White | 68.6 (5099) | Ref |  |
| Any other | 71.5 (4181) | **0.81 (0.75-0.89)** | **<0.001** |
| **Country** |  |  |  |
| England | 65.5 (2725) | Ref |  |
| Canada | 73.0 (3206) | **1.42 (1.28-1.58)** | **<0.001** |
| US | 71.0 (3349) | **1.20 (1.08-1.34)** | **<0.001** |
| **Vaping status** |  |  |  |
| Never vaped | 84.8 (6744) | Ref |  |
| Former/experimental vaping | 52.3 (2314) | **0.32 (0.29-0.36)** | **<0.001** |
| Vaped in past 30 days | 24.6 (222) | **0.11 (0.09-0.13)** | **<0.001** |
| **Smoking status** |  |  |  |
| Never smoked | 82.5 (7345) | Ref |  |
| Former/experimental smoking | 45.9 (1735) | **0.34 (0.31-0.38)** | **<0.001** |
| Smoked in past 30 days | 34.1 (200) | **0.26 (0.21-0.32)** | **<0.001** |
| **Packaging * Nicotine condition** † |  |  | *χ^2^*(2) = 0.41, p = 0.82 |

* This regression is similar to a regression in *Table 2* in the manuscript except for the vaping and smoking status variables that were included here separately compared with a combined vaping/smoking variable in the regression in *Table 2*.

† The interaction term between packaging and nicotine conditions was non-significant and was removed from the final model.

Supplement table 2 Multinomial regression model for selecting ‘Don’t know’ or ‘I have no interest in trying any of these products’ compared with ‘Any of e-liquids’ adjusted for sociodemographic, vaping and smoking, and intervention variables (n=13426).

|  | **Any of e-liquids (reference category)** | **Don’t know** | | | **I have no interest in trying any of these products** | | |
| --- | --- | --- | --- | --- | --- | --- | --- |
|  | **% (n)** | **% (n)** | **aOR (95% CI)** | **p value** | **% (n)** | **aOR (95% CI)** | **p value** |
| **Intercept** |  |  | **0.21 (0.14-0.31)** | **<0.001** |  | **11.4 (9.65-13.60)** | **<0.001** |
| **Packaging condition** |  |  |  |  |  |  |  |
| Branded | 33.0 (1479) | 2.0 (90) | Ref |  | 65.0 (2912) | Ref |  |
| Standardised white | 26.0 (1162) | 2.9 (131) | **1.99 (1.50-2.64)** | **<0.001** | 71.0 (3171) | **1.58 (1.43-1.76)** | **<0.001** |
| Standardised olive | 24.6 (1101) | 2.5 (114) | **1.83 (1.37-2.44)** | **<0.001** | 72.9 (3266) | **1.71 (1.54-1.91)** | **<0.001** |
| **Nicotine condition** |  |  |  |  |  |  |  |
| Low (3 mg/mL) | 27.8 (1866) | 2.4 (162) | Ref |  | 69.8 (4686) | Ref |  |
| High (20 or 59 mg/mL) | 27.9 (1876) | 2.6 (173) | 1.04 (0.83-1.31) | 0.72 | 69.5 (4663) | 0.95 (0.88-1.04) | 0.29 |
| **Sex** |  |  |  |  |  |  |  |
| Female | 29.0 (2681) | 2.2 (203) | Ref |  | 68.8 (6374) | Ref |  |
| Male | 25.5 (1061) | 3.2 (132) | **1.66 (1.31-2.10)** | **<0.001** | 71.4 (2975) | **1.21 (1.10-1.33)** | **<0.001** |
| **Age (years)** |  |  |  |  |  |  |  |
| 16 | 20.9 (543) | 2.9 (76) | Ref |  | 76.1 (1976) | Ref |  |
| 17 | 23.5 (751) | 2.4 (77) | 0.79 (0.56-1.11) | 0.174 | 74.0 (2361) | 0.98 (0.85-1.13) | 0.75 |
| 18 | 30.9 (1371) | 2.5 (112) | **0.69 (0.51-0.94)** | **0.020** | 66.6 (2960) | **0.77 (0.68-0.88)** | **<0.001** |
| 19 | 33.7 (1077) | 2.2 (70) | **0.56 (0.40-0.79)** | **0.001** | 64.1 (2052) | **0.75 (0.65-0.86)** | **<0.001** |
| **Race/ethnicity** |  |  |  |  |  |  |  |
| White | 29.2 (2201) | 2.4 (183) | Ref |  | 68.3 (5142) | Ref |  |
| Any other | 26.1 (1541) | 2.6 (152) | 1.04 (0.82-1.31) | 0.76 | 71.3 (4207) | **0.83 (0.76-0.91)** | **<0.001** |
| **Country** |  |  |  |  |  |  |  |
| England | 32.5 (1369) | 2.4 (99) | Ref |  | 65.1 (2740) | Ref |  |
| Canada | 24.7 (1098) | 2.6 (115) | **1.43 (1.08-1.91)** | **0.013** | 72.7 (3234) | **1.48 (1.33-1.65)** | **<0.001** |
| US | 26.7 (1275) | 2.5 (121) | 1.31 (0.99-1.75) | 0.059 | 70.7 (3375) | **1.25 (1.13-1.40)** | **<0.001** |
| **Smoking/vaping status** |  |  |  |  |  |  |  |
| Never used | 10.8 (755) | 1.9 (33) | Ref |  | 87.3 (6116) | Ref |  |
| Former vaping/smoking | 34.9 (1362) | 2.7 (105) | **0.44 (0.34-0.59)** | **<0.001** | 62.4 (2437) | **0.22 (0.20-0.24)** | **<0.001** |
| Vaped in past 30 days | 65.0 (1247) | 4.1 (78) | **0.36 (0.27-0.48)** | **<0.001** | 30.9 (592) | **0.06 (0.05-0.06)** | **<0.001** |
| Vaped and smoked in past 30 days | 75.2 (258) | 2.3 (8) | **0.18 (0.08-0.37)** | **<0.001** | 22.4 (77) | **0.04 (0.03-0.05)** | **<0.001** |
| Smoked in past 30 days | 46.5 (120) | 4.3 (11) | 0.56 (0.29-1.07) | 0.079 | 49.2 (127) | **0.13 (0.10-0.17)** | **<0.001** |
| **Packaging * Nicotine condition** † |  |  |  |  |  |  | *χ^2^*(4) = 1.14, p = 0.89 |

† The interaction term between packaging and nicotine conditions was non-significant and was removed from the final model.

Supplement table 3 Sample characteristics by participants’ perceived harm of e-liquid products including ‘Don’t know’ response (*n* = 13755).

|  | **Not at all harmful** | **Less harmful than smoking** | **As harmful as smoking** | **More harmful than smoking** | **Don’t know** | **Refused** |
| --- | --- | --- | --- | --- | --- | --- |
| Total | 2.1% (290) | 31.6 (4346) | 43.2 (5957) | 10.6 (1461) | 12.4 (1701) | 0.3 (46) |
| **Packaging condition** |  |  |  |  |  |  |
| Branded | 2.8 (129) | 34.3 (1579) | 40.8 (1878) | 9.7 (445) | 12.1 (555) | 0.3 (14) |
| Standardised white | 1.7 (76) | 30.7 (1415) | 44.8 (2062) | 10.7 (491) | 11.7 (539) | 0.5 (23) |
| Standardised olive | 1.8 (85) | 29.4 (1352) | 43.9 (2017) | 11.4 (525) | 13.2 (607) | 0.2 (9) |
| **Nicotine condition** |  |  |  |  |  |  |
| Low (3 mg/mL) | 2.3 (161) | 32.3 (2223) | 42.3 (2915) | 10.4 (720) | 12.2 (844) | 0.4 (29) |
| High (20 or 59 mg/mL) | 1.9 (129) | 30.7 (2123) | 44.0 (3042) | 10.7 (741) | 12.4 (857) | 0.2 (17) |
| **Sex** |  |  |  |  |  |  |
| Male | 2.9 (125) | 33.3 (1432) | 40.3 (1732) | 9.1 (390) | 14.1 (606) | 0.4 (17) |
| Female | 1.7 (165) | 30.7 (2914) | 44.5 (4225) | 11.3 (1071) | 11.5 (1095) | 0.3 (29) |
| **Age (years)** |  |  |  |  |  |  |
| 16 | 1.9 (50) | 28.8 (771) | 44.5 (1193) | 10.4 (278) | 14.1 (378) | 0.4 (11) |
| 17 | 2.1 (69) | 30.3 (993) | 42.7 (1402) | 10.5 (345) | 14.2 (465) | 0.2 (8) |
| 18 | 2.3 (105) | 32.9 (1497) | 42.6 (1942) | 10.9 (497) | 11.0 (501) | 0.3 (14) |
| 19 | 2.0 (66) | 33.1 (1085) | 43.3 (1420) | 10.4 (341) | 10.9 (357) | 0.4 (13) |
| **Race/ethnicity** |  |  |  |  |  |  |
| White | 2.2 (165) | 34.3 (2606) | 42.8 (3249) | 8.8 (665) | 11.6 (879) | 0.3 (25) |
| Any other | 1.8 (110) | 28.4 (1697) | 44.0 (2627) | 12.9 (769) | 12.5 (746) | 0.3 (16) |
| **Country** |  |  |  |  |  |  |
| England | 2.6 (114) | 42.8 (1847) | 35.9 (1549) | 6.0 (261) | 12.4 (536) | 0.2 (9) |
| Canada | 1.9 (88) | 28.1 (1296) | 45.0 (2073) | 11.2 (514) | 13.3 (614) | 0.4 (19) |
| US | 1.8 (88) | 24.6 (1203) | 47.8 (2335) | 14.1 (686) | 11.3 (551) | 0.4 (18) |
| **Vaping/smoking status** |  |  |  |  |  |  |
| Never used | 1.1 (76) | 25.1 (1812) | 47.5 (3431) | 12.3 (892) | 13.9 (1002) | 0.2 (17) |
| Former vaping/smoking | 1.7 (68) | 33.9 (1348) | 43.7 (1737) | 9.9 (392) | 10.5 (416) | 0.4 (16) |
| Vaped in past 30 days | 5.5 (107) | 47.5 (927) | 30.2 (589) | 6.5 (126) | 10.0 (195) | 0.4 (16) |
| Vaped and smoked in past 30 days | 8.3 (29) | 49.0 (172) | 27.6 (97) | 5.4 (19) | 8.5 (30) | 1.1 (4) |
| Smoked in past 30 days | 3.4 (9) | 30.5 (80) | 35.1 (92) | 12.2 (32) | 18.3 (48) | 0.4 (1) |

Supplement table 4 Multinomial regression model predicting 'Not at all harmful' and ‘As harmful/more harmful/Don’t know’ responses versus 'Less harmful than smoking cigarettes' response as a reference, adjusted for demographic, vaping/smoking, packaging and nicotine conditions and the interaction between vaping/smoking status and packaging conditions.

|  | **Not at all harmful** | | **As harmful/more harmful/Don’t know** | |
| --- | --- | --- | --- | --- |
|  | **aOR (95% CI)** | **p value** | **aOR (95% CI)** | **p value** |
| Intercept | **0.18 (0.11-0.30)** | **<0.001** | 1.11 (0.91-1.36) | 0.30 |
| **Packaging condition** |  |  |  |  |
| Branded | Ref |  | Ref |  |
| Standardised white | 0.64 (0.39-1.08) | 0.093 | 0.92 (0.73-1.16) | 0.50 |
| Standardised olive | 0.87 (0.54-1.39) | 0.55 | 0.95 (0.75-1.19) | 0.63 |
| **Nicotine condition** |  |  |  |  |
| Low (3 mg/mL) | Ref |  | Ref |  |
| High (20 or 59 mg/mL) | 0.87 (0.68-1.11) | 0.25 | 1.08 (0.997-1.16) | 0.059 |
| **Sex** |  |  |  |  |
| Male | Ref |  | Ref |  |
| Female | **0.63 (0.49-0.81)** | **<0.001** | **1.13 (1.05-1.23)** | **0.003** |
| **Age (years)** |  |  |  |  |
| 16 | Ref |  | Ref |  |
| 17 | 1.05 (0.71-1.55) | 0.77 | 0.94 (0.84-1.06) | 0.30 |
| 18 | 1.00 (0.69-1.44) | 0.99 | **0.88 (0.79-0.98)** | **0.020** |
| 19 | 0.80 (0.53-1.19) | 0.27 | **0.86 (0.76-0.96)** | **0.010** |
| **Race/ethnicity** |  |  |  |  |
| White | Ref |  | Ref |  |
| Any other | 1.22 (0.94-1.58) | 0.134 | 1.08 (0.995-1.16) | 0.066 |
| **Country** |  |  |  |  |
| England | Ref |  | Ref |  |
| Canada | 1.02 (0.76-1.39) | 0.88 | **1.93 (1.76-2.12)** | **<0.001** |
| US | 1.27 (0.94-1.72) | 0.114 | **2.28 (2.08-2.51)** | **<0.001** |
| **Vaping/smoking status** |  |  |  |  |
| Never used | **0.28 (0.17-0.47)** | **<0.001** | **2.37 (1.96-2.85)** | **<0.001** |
| Former vaping/smoking | **0.54 (0.34-0.87)** | **0.011** | **1.58 (1.29-1.93)** | **<0.001** |
| Vaped in past 30 days | Ref |  | Ref |  |
| Vaped and smoked in past 30 days | 1.43 (0.69-2.98) | 0.34 | 0.84 (0.54-1.29)) | 0.42 |
| Smoked in past 30 days | 1.81 (0.74-4.46) | 0.195 | **2.46 (1.50-4.02)** | **<0.001** |
| **Interactions^¥^** |  |  |  |  |
| Never used : Standardised white | 1.70 (0.80-3.62) | 0.167 | **1.40 (1.07-1.82)** | **0.014** |
| Former vaping/smoking : Standardised white | 0.57 (0.25-1.31) | 0.186 | **1.43 (1.08-1.90)** | **0.013** |
| Vaped and smoked in past 30 days : Standardised white | 0.67 (0.19-2.29) | 0.52 | 1.16 (0.64-2.12) | 0.63 |
| Smoked in past 30 days : Standardised white | 0.20 (0.02-1.84) | 0.155 | 1.10 (0.56-2.17) | 0.77 |
| Never used : Standardised olive | 0.72 (0.32-1.62) | 0.43 | **1.44 (1.11-1.88)** | **0.006** |
| Former vaping/smoking : Standardised olive | 0.71 (0.34-1.49) | 0.37 | **1.42 (1.07-1.89)** | **0.015** |
| Vaped and smoked in past 30 days : Standardised olive | 1.32 (0.47-3.71) | 0.60 | 1.51 (0.82-2.78) | 0.184 |
| Smoked in past 30 days : Standardised olive | — | — | 1.59 (0.76-3.33) | 0.22 |

— None of youth who smoked in the past 30 days chose ‘Not at all harmful’ response—ORs could not be calculated.

¥ Odds ratios for interaction terms represent adjustments associated with using different packaging conditions than the one which was used for main effects (i.e., branded e-liquid packs). For instance, compared with those who vaped in past 30 days, those who never used were 2.37 times more likely to select ‘as harmful/more harmful/don’t know’ over ‘less harmful than smoking cigarettes’ when assessing branded e-liquid packs. To assess the odds that those who never used (compared with those who vaped in past 30 days) selected ‘as harmful/more harmful/don’t know’ over ‘less harmful than smoking cigarettes’ when assessing standardised white e-liquid packs, the main effects odds ratio (2.37) has to be multiplied by the interaction term of ‘never user : standardised white odds ratio’ (1.40): 2.37 * 1.40 = 3.318. Odds ratios for different reference pack conditions are shown in Supplement figure 1.

Supplement figure 1 Odds ratios of youth in different vaping/smoking groups perceiving different e-liquid pack designs as ‘as harmful/more harmful/don’t know’ over ‘less harmful than smoking cigarettes’. Reference group was past 30-day vapers (a vertical dashed line for odds ratio 1.0). Asterisks (*) show odds ratios where interactions between vaping/smoking status and packaging conditions were statistically significant.
